# Proteomic investigation of signaling dynamics: from static maps to network rewiring

**DOI:** 10.1042/BSR20250116

**Published:** 2026-07-16

**Authors:** Ugo Dionne, Kosar Vafaee, Geoffrey G. Hesketh, Anne-Claude Gingras

**Affiliations:** 1 Lunenfeld-Tanenbaum Research Institute, Sinai Health, Mount Sinai Hospital, Toronto, Ontario, Canada; 2 Department of Molecular Genetics, University of Toronto, Toronto, Ontario, Canada

**Keywords:** cancer, mass spectrometry, phoshorylation, protein dynamics, proteomics, signalling

## Abstract

Cellular processes are controlled by interconnected networks of protein–protein interactions that can be dynamically regulated by post-translational modifications such as phosphorylation. Dysregulation of signaling pathways can drive cellular transformation and contribute to cancer treatment resistance. Mass spectrometry (MS)-based approaches have emerged as key technologies to study both protein function and their dynamic regulation at a network level. Modern proteomics allows investigators to study how signaling networks are rewired in response to genetic lesions, external cues, and targeted therapies, enabling the comparison of baseline (steady-state) networks to perturbed states. Here, we briefly describe key advancements in proteomics to study signaling dynamics, including affinity-purification combined with MS, proximity proteomics (e.g., BioID, APEX), and phosphoproteomics. We highlight how proteomics has led to the identification of comprehensive protein–protein interaction networks, to the delineation of protein subcellular localization maps and to discoveries regarding their dynamics and rewiring in disease. Finally, we comment on the future directions of proteomics to study signaling dynamics, enabled by next-generation MS instruments and AI-driven data analysis, and discuss how these developments are paving the way for clinical translation by bringing quantitative network biology into patient-relevant contexts.

The complexity of cellular processes arises from their vast and interconnected regulatory networks. While the human genome encodes roughly 20,000 genes, our cells potentially express between 100,000 and 1,000,000 proteins, known as proteoforms [1]. This molecular diversity stems from our cells’ ability to modify their proteins via dynamic regulatory mechanisms, ranging from alternative splicing to post-translational modifications (PTMs). These changes can alter a proteoform’s interactions with other molecules, its subcellular localization, its catalytic activity, and its structure [2–6]. Such proteoform modulation allows cells to change their behavior in response to cues on time scales ranging from seconds to hours. For example, extracellular signals detected by receptor tyrosine kinases (RTKs) rapidly trigger the assembly of intracellular complexes and increases in protein phosphorylation [7,8]. Therefore, understanding protein function requires systems-level investigation of their dynamic regulation.

Protein phosphorylation is a prominent cellular regulatory mechanism. This PTM acts as a molecular switch that can alter the functions of proteins, including their protein–protein interactions (PPIs), stability, localization, and enzymatic activity [5,9–11]. Importantly, the formation and disassembly of dynamic cellular structures such as membraneless organelles are also regulated by kinases, phosphatases, and protein phosphorylation [12–20]. However, major challenges remain: the functional consequences of most of the thousands of phosphosites that have been identified, and the kinases responsible, remain unknown [11,21]. Filling this gap in knowledge is critical because the disruption of PPI and phosphorylation networks is a frequent driver of cancer following genetic aberrations, such as point mutations, deletions, amplifications, and genomic rearrangements [2,22–26]. Effectors of phosphorylation-dependent signaling pathways are particularly affected and disease-associated mutations are predicted to be enriched at PPI interfaces [27]. These observations reinforce the view that disease phenotypes often emerge from multi-layered changes to signaling transduction networks, and not only from changes in protein expression level. Cancer, in addition to being a genetic and epigenetic condition, is also a disease of signaling dysregulation. As such, many kinases, especially tyrosine kinases, often function as oncogenes and their counteracting regulators as tumor suppressors. Accordingly, the two most frequent signaling alterations in cancers are the upregulation of RTK–RAS–MAPK and PI3K–AKT pathways [28–31]. Since the first developed kinase inhibitor in 2001 targeting the tyrosine kinase fusion BCR::ABL1, close to 100 drugs targeting kinases have been approved by the FDA [32–34]. Importantly, kinase inhibitor treatments almost always lead to the emergence of resistance mechanisms, linked directly to the ability of our cells to rewire their signaling networks to bypass treatments [32,35–38]. It is therefore imperative to study the plasticity of signaling pathways as this is fundamentally linked to protein function and diseases such as cancer [39]. A central question is thus how signaling networks are wired in space and time, and how this wiring is reshaped in response to perturbations.

Mass spectrometry (MS) based proteomics has emerged as a key technology used to study cellular signaling dynamics in normal and cancer cells [3,40–46]. The convergence of more sensitive instrumentation, improved sample preparation, and computational advances now enables comparisons across time, perturbations, and cell states, revolutionizing the way research teams use proteomics to investigate the dynamic regulation of proteins. In the present mini-review, we briefly recapitulate key advancements and describe examples of seminal discoveries regarding the dynamic interconnectivity of proteins and their dysregulation in diseases, with an emphasis on PPIs, phosphorylation, and emerging technologies.

## Proteomics to study protein interactions and phosphorylation

Several early landmark protein interactions studies used non-MS approaches such as yeast-two-hybrid to identify binary PPIs across model organisms [47–51]. Affinity-purification combined with mass spectrometry (AP-MS) [52,53] however soon became a cornerstone for studying soluble protein complexes, complemented by other approaches including co-fractionation combined with MS [54–56] to look at both protein complexes and subcellular localization [57–62]. In AP-MS, by enriching a protein of interest (aka ‘bait’) through the use of affinity-based tags or antibodies, the stable binding partners (aka ‘preys’) can be identified by MS. This was first showcased with the discovery of hundreds of PPIs in *Escherichia coli* [63] and *Saccharomyces cerevisiae* [64–69]. AP-MS was then extended to characterizing non-soluble complexes including membrane-bound proteins [70] and to other model organisms [71]. Large-scale efforts have been applied to the discovery of PPIs for most human proteins by AP-MS [72,73], including the BioPlex Network, OpenCell, and others [62,74–78]. New AP-MS strategies continue to be developed, such as deep interactome profiling by mass spectrometry, which incorporates deep learning signal processing [79]. Importantly, pull-downs from cellular lysates have been extended to chemical biology with, for example, kinobeads, where kinase inhibitors are immobilized to a matrix in order to identify both ON- and OFF-targets via MS approaches [80–83], bridging target discovery with mechanism-of-action studies.

### Establishment of proximity proteomics as an orthogonal approach

A limitation of AP-MS is the requirement to maintain PPIs during lysis and purification, which can lead to the loss of lower affinity interactions and of proteins localized to less soluble cellular environments, such as lipid membranes. Of note, several non-MS based approaches, for example protein complementation assays, have been developed to study PPIs between membrane-localized proteins [51,84–87]. These challenges were mitigated by the introduction of proximity proteomics, which includes proximity-dependent biotinylation (PDB) methods such as BioID and APEX [88–93] that are used to explore the protein neighbourhood of a bait of interest, revealing its potential PPIs and subcellular localization [94–99]. In PDB, baits are fused to a biotin ligase or peroxidase and prey proteins are covalently labeled with reactive biotin moieties in living cells. Biotinylated preys are then enriched by taking advantage of the extremely strong natural streptavidin–biotin interaction [100]. This allows harsh lysis under strong solubilizing conditions, allowing the identification of insoluble proteins and residents of less-stable structures, such as membraneless organelles [101–105]. This strategy has enabled the establishment of protein ‘proximity maps’ of organelles and human cells [99,105–107], and the profiling of complete families of proteins [108,109]. In addition, the development of split versions of the PDB enzymes potentially increases the resolution of proximity proteomics and subcellular mapping [110–113]. AP-MS and BioID have also been combined experimentally to generate comprehensive lists of protein interactions [93,114]. The generation of large amounts of protein–protein association data highlighted the need to develop probabilistic scoring algorithms that help identify ‘true’ PPIs, such as Significance Analysis of INTeractome (SAINT), Comparative Proteomic Analysis Software Suite (CompPASS), and the contaminant repository CRAPome [115–120]. These PPIs have been incorporated in large databases [121–125], such as BioGRID and IntAct [126–128], which represent useful resources [129].

Although they each have their strengths and disadvantages, AP-MS and BioID are highly complementary approaches [88]. AP-MS has been widely used in the field, including through the BioPlex project, to detect and quantify soluble protein complex components, whereas BioID is particularly applicable for defining subcellular localization and identifying protein–protein associations from less-soluble cellular structures. In addition, crosslinking-MS can add structural resolution to the identification of PPIs, for instance to resolve the subunit organization of protein complexes [130,131].

### Development of phosphopeptide enrichment strategies and their applications

Proteomic investigation of phosphorylation required overcoming its low stoichiometric abundance, which was made possible by the establishment of phosphopeptide enrichment strategies. While multiple approaches have been employed for enrichment [132], the most common is immobilized metal-affinity chromatography (IMAC), first developed using Fe3+ and now more commonly performed using Ti4+ (TiO2) and Zr4+ (ZrO2) [133–136]. The combination of IMAC with strong cation exchange chromatography led to major increases in phosphopeptide identification [137–140]. Importantly, the development of computational statistical methods resulted in standardized scoring systems for localizing phosphorylation sites [141,142]. Although phosphotyrosines are by far the least abundant phosphopeptides at steady state, they are important signal mediators in normal and cancer cells. Because IMAC-based datasets are dominated by phosphoserine and phosphothreonine peptides, antibody-based and domain engineering approaches (e.g., SH2 superbinders) were developed to specifically enrich phosphotyrosine peptides [143–152]. The incredible promise of phosphoproteomics and phosphotyrosine enrichment was highlighted by an early study using lung cancer cell lines and tissues, which led to the identification of ALK and ROS1 fusions [153]. Phosphoproteomics strategies are now widely used to study signaling pathways and how they are altered in diseases, with tens of thousands of phosphorylation sites now identified and evidence suggesting that nearly all human proteins can be modified by this PTM [154–159]. Databases containing phosphosites that have been detected by MS are available, including PhosphoSitePlus that also incorporates the recognized motifs of most human kinases and supports motif-based prediction of kinase–substrate relationships, although it has been suggested that some phosphosites reported are likely false-positives [160–163] (Table 1). Importantly, tools are also being developed to visualize and interpret phosphoproteomics data, such as PTMNavigator, which is integrated into ProteomicsDB [164–166] (Table 1). Notably, recent efforts have highlighted the existence of phosphorylation on other amino acids, such as pHis, pLys and pArg residues. Technologies are being developed to specifically detect and study these modifications, and it will be interesting to determine their dynamic nature in different cellular states and following perturbations [11,167,168]. The establishment of MS as a powerful method to delineate the subcellular localization of proteins, to detect and quantify their PPIs and phosphorylation sites ultimately paved the way for the use of proteomics to investigate dynamic and context-dependent signaling.

**Table 1:** Databases and tools for the analysis of interactomics and phosphoproteomics datasets

MS-based strategy	Databases	Tools	Description
Interactomics	BioGRID [126,127] IntAct [128] Cell map [106] STRING [122–125] OpenCell [78]	FragPipe-Analyst [291–293]	Usually in combination with FragPipe [291–293] or DIA-NN [269] to visualize and analyze proteomics data.
		Spectronaut [267]	For DIA data, also allows visualization and analysis.
		ProHits-viz [294]	Visualization and analysis tool, used mostly in combination with SAINT [115–118].
		SAINT [115–118]	A probabilistic algorithm that allows the identification of ‘true’ interactors, using user-defined negative controls.
		MSstats [295]	Analysis tool which supports DDA, DIA, and TMT quantification and is compatible with FragPipe [291–293].
		Perseus [298–299]	Normally used in combination with MaxQuant [296,297] for data visualization.
Phosphoproteomics	PhosphoSitePlus [160] UniProt [301]	FragPipe-Analyst [291–293]	Usually in combination with FragPipe [291–293] or DIA-NN [269] to visualize and analyze proteomics data.
		Spectronaut [267]	For DIA data, also allows visualization and analysis.
		MSstatsPTM [271]	A variation of MSstats [295] for PTM analysis.
		PTM Navigator [164]	Visualization and analysis tool centered around signaling pathways.
		Phospho-Analyst [300]	Normally used in combination with MaxQuant [296,297] for data visualization and analysis.

## From steady-state to the dynamic regulation of proteins

Over the past 15 years, hundreds of published research papers have used proteomics to investigate how PPI and phosphorylation networks change dynamically following perturbations and in the context of disease. In this section, we will describe key examples of how new MS-based approaches and analysis strategies have led to the discovery of impactful biological findings. Note that several structural proteomics developments, including crosslinking MS and limited proteolysis, have also enabled studying how the structure of proteins and their PPIs change in dynamic and disease states [130,131,169–175]. Importantly, crosslinking approaches, including photo-crosslinking with genetically encoded unnatural amino acids, can add structural resolution to the study of PPIs [176].

### Combination of AP-MS with perturbations to study dynamic regulation

Building on pioneering work using AP-MS to study the dynamic rewiring of PPIs following perturbations (such as EGF and TNF-α) [144,177–179], proteomics has been used widely to investigate the dynamic nature of PPI networks. Landmark studies combined AP-MS with targeted quantitative MS approaches (specifying peptides to be quantified) to delineate changes in the PPIs of the adaptor GRB2 and scaffold SHC1 in response to different growth factors such as EGF [180,181]. These studies illustrated that AP-MS can be used to quantitatively delineate the dynamic nature of signaling networks in a time-resolved manner by demonstrating that protein complex composition changes in the scale of seconds to minutes. The same approach was then used to investigate phosphotyrosine-dependent PPIs and uncovered a negative regulatory feedback mechanism involving RTKs and proteins comprising SRC homology 3 (SH3) domains [182]. An extension to the approach combining AP-MS and data independent acquisition (DIA) [183,184] enabled the investigation of the rewiring of PPI networks following cell stimulation (IGF-1), drug treatment (HSP90 inhibitor), and cancer mutations. DIA-based AP-MS was subsequently applied to primary T cells [185], to profile the rewiring of bromo-and-extra-terminal (BET) PPIs following treatment with a pan-BET BRD inhibitor [186], and to investigate the importance of the position of PPI domains in adaptor proteins [187]. Additionally, remodeling of the PPIs of ∆F508 CFTR in the context of cystic fibrosis was studied by quantitative AP-MS, which increased the understanding of this disease at a molecular level [188].

### Improvements of PDB approaches allow dynamic studies

Proximity proteomics has also been increasingly applied to dynamic studies, especially since the development of new generations of APEX (APEX2) [92] and biotin ligases (TurboID and miniTurbo) [189] (Table 2). Moreover, new biotin ligases are frequently being engineered (e.g., ultraID and PhastID) [190,191] and PDB has been optimized and combined with other tools to allow the users to control their activity [110–113,192–195] (Table 2). In 2017, two studies used APEX to profile GPCRs and spatiotemporally delineate their protein complexes, demonstrating the potential of this technology for dynamic signaling studies [196,197]. Soon after, another use of proximity proteomics was showcased by two publications that used BioID and APEX approaches to profile the dynamic changes in the composition of membraneless organelles, including RNA bodies and stress granules [101,103]. Both studies led to the discovery of pre-assembled protein associations, before the existence of stress stimuli. BioID was also used to profile different components of the endo-lysosome fusion machinery as organelle sensors, which led to the identification of a new mechanism of nutrient-dependent mTORC1 activation [198]. Recently, APEX was also used to spatiotemporally profile the activated μ-opioid GPCR [199]. Additionally, efforts from many research groups have led to the optimization of PDB techniques, including singlet oxygen generators, to map the extracellular protein portions of the plasma membrane and how it changes dynamically [200–205]. This is motivated not only by discovery research but also by the important clinical potential of targeting proteins that are specifically localized at the surface of malignant cells.

**Table 2:** Different enzymes and their characteristics for PDB approaches

PDB methods	Origin	PDB enzymes	Size	Labeling time
Peroxidases	*Armoracia rusticana* (Horseradish)	HRP [100,304]	34 kDa	Seconds–1 min
	Pea or Soybean ascorbate peroxidases	APEX/APEX2 [91]	28 kDa	Seconds–1 min
Biotin ligases	*Escherichia coli*	BioID (BirA*) [89]	35 kDa	6–24 h
		TurboID [189]	35 kDa	10–60 min
		miniTurbo [189]	28 kDa	10–60 min
	*Aquifex aeolicus*	BioID2 [302]	27 kDa	6–24 h
		ultraID [190]	19 kDa	10–30 min
	*Bacillus subtilis*	BASU [303]	35 kDa	Minutes to Hours
	*Pyrococcus horikoshii*	PhastID [191]	27 kDa	1–10 min

### Phosphoproteomics to investigate signal rewiring

The innovative studies that led to the development of phosphopeptide enrichment technologies were also in many cases the first demonstrations of phosphoproteomics to study signaling dynamics, again using EGF treatments as a proof of concept and for discovery [138,143,206]. Phosphoproteomics strategies were then rapidly applied to delineate phosphoproteins across the cell cycle [207–210], to study the effects of kinase deletions in yeast [211] and of growth factors and kinase inhibitors in cancer cells [212,213]. Three studies then used this approach to investigate mTOR signaling in response to insulin with or without drug treatments, which led to the identification of the new mTORC1 effector GRB10 and of functional links between mTORC2 and AKT [214–216]. Moreover, proteomics approaches led to the discovery that mTORC1-dependent signaling controls the *de novo* pyrimidine biosynthesis pathway metabolic flux through phosphorylation of CAD [217,218]. Phosphoproteomics studies also led to a better understanding of the molecular basis of cellular growth, autophagy, and nutrient sensing control via the kinases mTOR, ULK1/2, GCN2, and AMPK [219–223]. Similar frameworks, combining growth factor treatments and inhibitors, were used to decipher RTKs, PTPN11 (SHP2) signaling, and chromatin remodeling [224–226]. A landmark study painted a comprehensive picture of the landscape of phosphorylation in cancer cells, unveiling important fundamental differences between phosphotyrosine and phosphoserine/threonine signaling [154]. In addition, a recent study built a gold-standard proteomics and phosphoproteomics Cell Cycle Database (CCdb) from non-transformed cells [227], providing a reference for evaluating aberrant signaling states in disease samples.

### *In vivo* phosphoproteomics in disease models

Numerous efforts established phosphoproteomics as a powerful tool for *in vivo* studies. Mouse models were used to analyze the dynamic signaling of the insulin response [228], adipocyte mitochondrial metabolism following time-restricted feeding [229], the circadian rhythm [230], nutrient sensing in mice liver mitochondria [231], brain GPCRs [232], and sleep deprivation [233]. A spatial dimension was added to the study of EGFR signaling in cells and mouse tissues by combining cell fractionation with phosphopeptide enrichment [234]. Recently, phosphoproteomics studies of dynamic signaling have been getting closer to the clinic. Phosphoproteomics was applied to human models of type 2 diabetes [235], to leukemia cells treated with lysine deacetylase inhibitors [236], to patient derived cell lines of ALK [237,238] or EGFR [239] driven lung cancers, with or without kinase inhibitor treatments and directly to patient biopsies to study exercise and insulin signaling [240]. These studies demonstrated the potential of phosphoproteomics to monitor the dynamic rewiring of signaling pathways, directly in samples relevant to patients where conventional molecular measurements lack functional resolution.

## Future directions

Since the turn of the millennium, proteomics has been increasingly used to study the functions of proteins and how they are dynamically regulated. From static to perturbation, time course and *in vivo* studies, proteomics has delineated the dynamic nature of signaling pathways, including how they are dysregulated in diseases such as cancer. Proteomics has enabled the identification of the PPIs of most human proteins and the establishment of proximity subcellular maps. It also led to a better understanding of the kinetics of complex assembly, of the different functional consequences of phosphotyrosine, -serine and -threonine modifications, of how kinases, especially tyrosine kinases, can drive cancer formation and respond to kinase inhibitor treatments and how proteins relocalize dynamically, including to membraneless organelles. The advent of next-generation MS platforms, including the Orbitrap Astral and timsTOF SCP/Ultra systems, is enabling high-speed, low-input phosphoproteomics and interactomics [241–243]. These now permit the quantification of signaling rewiring in scarce clinical samples, and in some cases at single-cell or spatial resolution, positioning proteomics to directly inform translational studies [244–254]. Importantly, single-cell and laser-capture microdissection MS-based approaches are quickly improving, with the identification of close to 5000 proteins per cell [248,255–258]. Two recent examples showcase the translational potential of these new generation instruments, by applying spatial proteomics to skin and liver disease samples [259,260]. Remarkably, this led to the discovery that inhibitors targeting the tyrosine kinase JAK can help cure toxic epidermal necrolysis [259].

As the sample input requirements decrease, the feasibility of sample preparation automation increases, with the potential of strongly improving reproducibility and throughput. Several efforts have established automated pipelines for phosphoproteomics [148,242,261,262], to identify protein expression changes following drug treatments [263], for the study of PPIs [243,264] and other MS-based dynamic studies [265]. DIA is being used more frequently for proteomics, especially for phosphoproteomics studies, including library free strategies such as direct DIA [261,266–272]. Recent benchmarking of common quantitative approaches suggested that DIA and isobaric tagging (e.g., TMT) are similar in their performance and are complementary for global proteomics performed on the Orbitrap Astral [273]. Phosphoproteomics using DIA can now be done routinely, making it an attractive alternative to isobaric labeling, depending on the number of samples to be analyzed jointly and peptide input amounts [274]. With increasing sensitivity and throughput comes an escalation in the amount and complexity of the data [275,276]. This necessitates a parallel revolution in data processing and analysis. Artificial intelligence (AI), including machine learning, is now applied to data processing, especially for DIA [266,269,275–277]. AI is also being used in the data analysis portions of proteomics and phosphoproteomics pipelines, with the goal of more efficiently extracting meaningful biological data [276,278]. Because of the lack of annotations for the functional impacts of phosphorylation on specific residues and of kinase–substrate relationships, it is still challenging to identify biologically meaningful information from dynamic quantitative studies. With newer technologies generating increasingly large datasets and high numbers of differential hits, the selection of candidates for validation and follow-up studies becomes more difficult. Although many analysis pipelines link proteomics data analysis to biological annotation tools such as Gene Ontology [279,280], incorporation with other datasets and insights, e.g., protein expression levels and kinase prediction tools [161,162], will improve the identification of potentially affected signaling pathways, although the integration of multi-omics datasets is still challenging. For instance, the differential expression of protein pairs has been used to study altered PPIs in disease [281]. Moreover, combining proteomics data analysis with other AI-based models, such as AlphaFold [282,283], which can help predict PPIs [284–287] or the structural effects of phosphosites and mutations [278,285,288–290], will drastically improve our ability to identify the most functionally relevant differential phosphosites and PPIs from MS-based studies.

**Figure 1:**
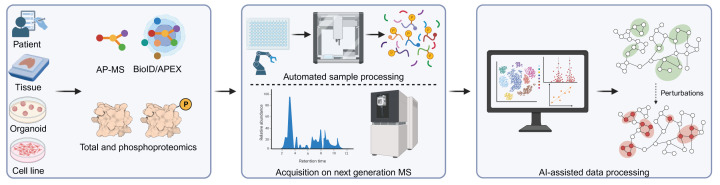
Next generation proteomics to study signaling dynamics A simplified representation of proteomic pipelines that are currently used to study the dynamic rewiring of signaling pathways following perturbations and in disease states. Limited samples from different origins are used for interactomics, total proteomics, and phosphoproteomics and processed in an automated manner. These samples are then analyzed using next-generation MS instruments. Finally, AI is used to process the raw data (e.g., for direct DIA) and help extract the most biologically relevant differential PPIs and phosphosites. This information can then be mapped at a network level to inform on potential targets for therapy in the context of diseases such as cancer. Created in BioRender. Dionne, U. (2026) https://BioRender.com/wckenho.

With all these recent developments, proteomic investigation of signaling dynamics can now be achieved with low sample input (e.g., patient-derived materials for spatial proteomics and single-cell analyses), automated sample processing, rapid and sensitive MS acquisition, and AI-assisted raw data analyses and biological interpretations (Figure 1). This will usher in a new era for the investigation of signaling dynamics via proteomic approaches, which has the promise of bridging the gap between fundamental molecular biology and personalized medicine.

## Abbreviations

AI: artificial intelligence
AP-MS: affinity-purification combined with mass spectrometry
BET: bromo-and-extra-terminal
DIA: data independent acquisition
EGF: epidermal growth factor
IMAC: immobilized metal-affinity chromatography
MS: mass spectrometry
PDB: proximity-dependent biotinylation
PPI: protein–protein interaction
PTM: post-translational modification
RTK: receptor tyrosine kinase
SAINT: Significance Analysis of INTeractome
SH2: SRC homolgy 2
